# Retraction: Severe contact dermatitis due to camomile: a common complementary remedy with potential sensitization risks

**DOI:** 10.1186/1710-1492-9-28

**Published:** 2013-08-12

**Authors:** Sibel Dogan

**Affiliations:** 1 Ankara Numune Training and Research Hospital, Clinic of Dermatology and Venerology, Ankara 06100, Turkey

## Retraction

This article [1] has been retracted by the authors. Although the patients originally gave consent to publication of their cases and images, they withdrew this consent shortly after publication. The article is no longer available online in order to protect patient confidentiality. The author apologizes for the inconvenience.
